# Untargeted GC-MS Metabolic Profiling of Anaerobic Gut Fungi Reveals Putative Terpenoids and Strain-Specific Metabolites

**DOI:** 10.3390/metabo15090578

**Published:** 2025-08-29

**Authors:** Lazarina V. Butkovich, Candice L. Swift, Chaevien S. Clendinen, Heather M. Olson, Samuel O. Purvine, Oliver B. Vining, Michelle A. O’Malley

**Affiliations:** 1 Department of Chemical Engineering, University of California, Santa Barbara, Santa Barbara, CA 93106, USA; 2 Earth and Biological Sciences Division, Pacific Northwest National Laboratory, Richland, WA 99352, USA; 3 Environmental Molecular Sciences Laboratory, Pacific Northwest National Laboratory, Richland, WA 99352, USA; 4 Institute for Collaborative Biotechnologies, University of California, Santa Barbara, CA 93106, USA

**Keywords:** anaerobic fungi, neocallimastigomycota, GC-MS, metabolomics, natural products

## Abstract

**Background/Objectives**: Anaerobic gut fungi (Neocallimastigomycota) are biotechnologically relevant, lignocellulose-degrading microbes with under-explored biosynthetic potential for secondary metabolites. Untargeted metabolomic profiling with gas chromatography–mass spectrometry (GC-MS) was applied to two gut fungal strains, *Anaeromyces robustus* and *Caecomyces churrovis*, to establish a foundational metabolomic dataset to identify metabolites and provide insights into gut fungal metabolic capabilities. **Methods**: Gut fungi were cultured anaerobically in rumen-fluid-based media with a soluble substrate (cellobiose), and metabolites were extracted using the Metabolite, Protein, and Lipid Extraction (MPLEx) method, enabling metabolomic and proteomic analysis from the same cell samples. Samples were derivatized and analyzed via GC-MS, followed by compound identification by spectral matching to reference databases, molecular networking, and statistical analyses. **Results**: Distinct metabolites were identified between *A. robustus* and *C. churrovis*, including 2,3-dihydroxyisovaleric acid produced by *A. robustus* and maltotriitol, maltotriose, and melibiose produced by *C. churrovis*. *C. churrovis* may polymerize maltotriose to form an extracellular polysaccharide, like pullulan. GC-MS profiling potentially captured sufficiently volatile products of proteomically detected, putative non-ribosomal peptide synthetases and polyketide synthases of *A. robustus* and *C. churrovis*. The triterpene squalene and triterpenoid tetrahymanol were putatively identified in *A. robustus* and *C. churrovis*. Their conserved, predicted biosynthetic genes—squalene synthase and squalene tetrahymanol cyclase—were identified in *A. robustus*, *C. churrovis*, and other anaerobic gut fungal genera. **Conclusions**: This study provides a foundational, untargeted metabolomic dataset to unmask gut fungal metabolic pathways and biosynthetic potential and to prioritize future efforts for compound isolation and identification.

## 1. Introduction

Anaerobic gut fungi (phylum Neocallimastigomycota) inhabit the rumens and hindguts of many mammalian and reptilian herbivores [1]. These gut fungi excel at breaking down lignocellulosic biomass and harbor a wealth of carbohydrate-active enzymes and predicted biosynthetic genes that synthesize secondary metabolites, exemplifying their biotechnological potential [2,3,4]. Despite the genetic potential of gut fungi, their secondary metabolism is largely uncharacterized. Previous metabolomic studies on gut fungi utilized untargeted liquid chromatography with tandem mass spectrometry (LC-MS/MS) to profile non-polar metabolites [2,5] and targeted gas chromatography with mass spectrometry (GC-MS) to profile lipids [6,7,8,9,10]. Expanding metabolomic datasets advance the understanding of anaerobic gut fungal metabolism. Existing datasets for anaerobic gut fungi lack untargeted GC-MS analysis with chemical derivatization, which detects small, volatile metabolites (<650 Da), including a set of metabolites missed by LC-MS/MS [11]. To address this gap in knowledge, we implemented the Metabolite, Protein, and Lipid Extraction (MPLEx) method for proteomic and untargeted GC-MS metabolomic analyses from the same gut fungal cell extractions.

In this study, we metabolically profiled two cultivable gut fungal strains with published reference genomes and distinct morphologies. The first strain, *Anaeromyces robustus* S4, is a polycentric fungus that forms rhizoids—root-like structures that penetrate substrates [3,12]. The second strain, *Caecomyces churrovis* A, is a monocentric fungus that instead forms bulbous holdfasts as a means to attach to surfaces and substrates [13,14]. To investigate gut fungal metabolites, we determined fungal products based on their detection in fungal cultures and absence in the rumen-fluid-based control media. Additionally, we determined metabolites that were likely acquired from the media and concentrated in fungal cells. We analyzed metabolites by two workflows. For the first workflow, we identified metabolites by comparison to NIST 14 and internal reference libraries. Using fatty acid profiling—a routine technique to taxonomically distinguish microbes [9,15,16]—we generated evidence that classifying gut fungal genera by fatty acid profiling is unreliable based on the inter-study variability in gut fungal fatty acid profiles [6]. For the second workflow, we performed exploratory analysis with the Global Natural Products Social Molecular Networking (GNPS) web platform [17,18], which revealed additional putative compound identifications, including for some secondary metabolites. We linked predicted biosynthetic genes for tetrahymanol, a triterpenoid sterol surrogate essential for gut fungal membranes [7,19,20], and its precursor squalene, and detected both compounds as gut fungal products in *A. robustus* and *C. churrovis*. Overall, the GC-MS profiling of *A. robustus* and *C. churrovis* provides a resource to better understand gut fungal metabolism and unlock their biotechnological potential.

## 2. Materials and Methods

### 2.1. Cultivation and Sample Preparation of Anaerobic Gut Fungi for GC-MS and Proteomics

*A. robustus* and *C. churrovis* were previously isolated via reed canary grass enrichment from the feces of sheep at the Santa Barbara Zoo [3,13]. The fungi were cultured and routinely passaged anaerobically in 9 mL of rumen-fluid-based Medium C or a reduced formulation of Medium C (0.025% *w*/*v* yeast extract (Thermo Fisher Scientific, Waltham, MA, USA), 0.05% *w*/*v* Bacto^TM^ Casitone (Thermo Fisher Scientific, Waltham, MA, USA), and 7.5% clarified rumen fluid) [21] with 0.1 g of switchgrass in a 10 mL Hungate tube. Cultures were passaged every 3–4 days by adding 1.0 mL inoculum to fresh media.

For each gut fungal strain, seed cultures were prepared by adding 1.0 mL of inoculum from routine passaging to 60 mL anaerobic serum bottles (VWR International, Radnor, PA, USA) with 40 mL of Medium C [21] and 5 g/L cellobiose (Thermo Fisher Scientific, Waltham, MA, USA). After incubation at 39 °C for 72 h (exponential growth phase), 1.0 mL of seed culture inoculum was used to inoculate a 100 mL anaerobic serum bottle (VWR International) with 80 mL of Medium C and 5 g/L cellobiose in quadruple replicates for each strain. Cultures were incubated at 39 °C for 72 h, and fungal mats were harvested in 50 mL Falcon tubes by centrifugation (3220× *g*, 4 °C, 10 min) with a swinging bucket rotor (Eppendorf^TM^ A-4-81, Framingham, MA, USA). Supernatants were removed, and each cell pellet was washed with 5.0 mL of sterile-filtered phosphate buffered saline (PBS) and transferred to 5 mL Eppendorf tubes. Quadruplicate samples for (i) Medium C with 5 g/L cellobiose and (ii) rumen fluid controls were similarly prepared for analysis. All samples were stored at −80 °C until they were shipped to the Pacific Northwest National Laboratory (PNNL) for MPLEx chemical extraction and data acquisition. The MPLEx method is a 3-in-1 method for the simultaneous extraction of metabolites, proteins, and lipids [22], and chemicals for the MPLEx method were acquired from Sigma-Alrich (St. Louis, MO, USA). Each sample was thawed on ice and extracted as described previously [2], except that the polar (top, aqueous) and non-polar (bottom, organic) layers of the triphasic MPLEx chemical extraction [22] were combined for GC-MS analysis. Proteins in the middle/interphase layer were used for proteomic analysis.

### 2.2. GC-MS Sample Derivatization and Data Acquisition

For all samples, dried metabolite extracts were derivatized using a modified version of the protocol used to create FiehnLib [11]. Samples underwent methoximation to protect carbonyl groups and reduce tautomeric isomers, followed by silylation with N-methyl-N-trimethylsilyltrifluoroacetamide (MSTFA) (Sigma-Aldrich) and 1% trimethylchlorosilane (Sigma-Aldrich) to derivatize hydroxy and amine groups to trimethylsilated (TMS) forms. GC-MS data were acquired for all randomized sample extracts, water blanks, and a standard mixture of C8–C28 fatty acid methyl esters (FAMEs) (Sigma-Aldrich). The FAME sample was used for retention index (RI) alignment to internal databases. An Agilent GC 7890A coupled with a single quadrupole MSD 5975C (Agilent Technologies, Santa Clara, CA, USA) was used to collect GC-MS data over a mass range of 50–550 *m*/*z*. The GC oven was held at 60 °C for 1 min after injection, followed by a temperature increase of 10 °C min^−1^ to a maximum of 325 °C and held for 5 min.

### 2.3. Identification of Known Compounds and Molecular Networking

For compound identifications using the NIST 14 GC-MS reference library and internal reference libraries (discussed in greater detail in the Supplementary Materials and Section 2), GC-MS raw data files were first processed using Metabolite Detector software, version 2.5 beta [23] (Supplementary Material 1). Compound identifications were defined as high confidence if they had a retention index match and >80% mass spectral match in an augmented version of the FiehnLib [24], containing spectra and retention indices for over 1200 metabolites (Supplementary Material 1). All matches were manually validated with NIST 14 or internal reference libraries. Compound identifications were defined as lower confidence if they had a retention index match and either a <80% spectral match or a >80% spectral match to multiple compounds, making definitive assignment difficult (Supplementary Material 1).

In parallel to the NIST-based workflow, a more exploratory analysis was also performed. Raw GC-MS .mzML files were processed with MS-DIAL (v4.9) [25] and uploaded to the GNPS (Global Natural Products Social Molecular Networking, http://gnps.ucsd.edu (accessed on 17 December 2024)) GC-MS EI data analysis workflow [17,18] for molecular networking and additional putative compound identifications via library search with mass spectral matching (Supplementary Material 2). Molecular networks were exported from GNPS and modified in Cytoscape (v3.10.3) [26]. An associated workflow (Python) for statistical analyses was made available on GitHub (https://github.com/O-Malley-Lab/GF_GCMS_data_analysis, accessed on 26 August 2025). The methods for molecular networking and data visualization are discussed in greater detail in the Supplementary Materials and Methods.

### 2.4. Proteomics Mass Spectrometry and Data Analysis

Proteomics data was acquired as described previously [2], with the following minor changes: MS spectra were acquired with a resolution of 70 k from 200 to 2000 *m*/*z* and an MS2 isolation window of 1.5 *m*/*z*. Proteomics mass spectrometry data were processed and analyzed as described previously [2,27], with the exception that peptide fragments were mapped to transcriptomes [3,13] translated into all open reading frames, as well as to biosynthetic genes predicted by antiSMASH v3.0 [28]. All MS/MS spectra were interrogated by a target–decoy approach using the database search tool MS-GF+ [29] with a ±20 ppm parent mass tolerance, no specific digestion enzyme settings, and a variable post-translational modification of oxidized methionine. Collated search results were combined and imported into a Microsoft SQL Server and filtered to a 1% false discovery rate (MS-GF+ Q-value). Unique peptides for each reference protein were counted, and peptide–spectrum matches for all peptides for a protein were counted (observation count values) (Supplementary Material 3 and 4).

## 3. Results and Discussion

### 3.1. Anaerobic Gut Fungi A. robustus and C. churrovis Produce Distinct Metabolites

GC-MS profiling of the anaerobic gut fungi *A. robustus* and *C. churrovis*, reveals insight into their core (primary) metabolism as well as their secondary metabolism. The fungi were cultivated on media with undefined rumen fluid that supports their laboratory cultivation. Due to the media complexity, media components were considered when interpreting the gut fungal GC-MS profiles. Metabolites were identified using two parallel approaches: (1) NIST 14 and internal reference libraries for characterized metabolites and (2) GNPS reference libraries for an exploratory analysis (see Section 2).

**Figure 1:**
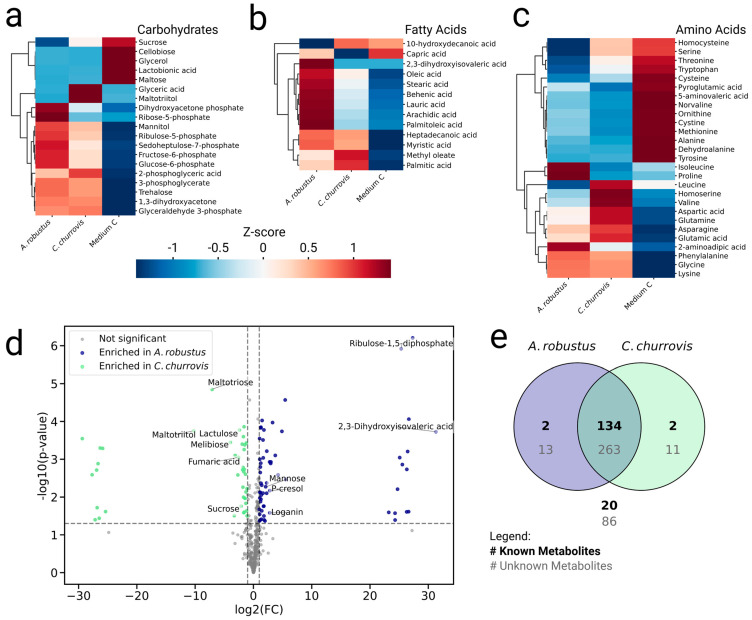
*A. robustus* and *C. churrovis* produced distinct metabolite profiles. Metabolites were identified by the retention index and mass spectral matching to NIST 14 and internal reference libraries. High-confidence compound identifications for (**a**) carbohydrates, (**b**) amino acids, and (**c**) fatty acids associate with sample type. (**d**) Metabolite features differentially detected between *A. robustus* and *C. churrovis* (|log2 fold-change| > 3, *p* < 0.05) are shown as labeled compounds. (**e**) Most metabolites (known and unknown) were detected (non-zero intensity in at least 2/4 biological replicates for a fungal strain) in both fungal strains, and a smaller set was uniquely detected in only one fungal strain. Created in BioRender. Butkovich, L. (2025) https://BioRender.com/u3vkl8o.

Comparing metabolite abundances between *A. robustus*, *C. churrovis*, and the media provides a starting point to generate hypotheses and explore metabolic trends (Figure 1). Probabilistic principal component analysis (pPCA) and Spearman rank correlations distinguished chemical profiles between gut fungi, rumen fluid, and Medium C (Figure S1). Between the two fungal strains, 11 known metabolites were differentially detected (|log2 fold-change| > 3 and *p*-value < 0.05), and 22 unknown metabolite features were highly differentially detected (|log2 fold-change| > 20 and *p*-value < 0.05) (Figure 1d). Most known and unknown metabolites were detected in both *A. robustus* and *C. churrovis* (non-zero intensity in at least two out of four biological replicates) (Figure 1e). By this definition for metabolite detection, 2 known metabolites (2,3-dihydroxyisovaleric acid and ribulose-1,5-diphosphate) and 13 unknown metabolites were detected in *A. robustus* and not *C. churrovis*, and 2 known metabolites (dihydrosphingosine and maltotriitol) and 11 unknown metabolites were detected in *C. churrovis* and not *A. robustus*.

The analysis of metabolite abundances across samples (*A. robustus*, *C. churrovis*, Medium C, and rumen fluid) distinguishes metabolites that are either (i) clearly produced by the gut fungi and not detected in the media or (ii) potentially acquired from the media (Figure S2). Among clear gut fungal products, several expected metabolites were identified, including dihydrosphingosine in *C. churrovis*, a key metabolite for sphingolipid biosynthesis [30], and succinic acid (succinate), a key product in gut fungal central metabolism [31,32]. Interestingly, *A. robustus* uniquely produced 2,3-dihydroxyisovaleric acid, an intermediate in branched-chain amino acid (isoleucine, leucine, and valine) biosynthesis, and this metabolite was absent in *C. churrovis* and the media.

Products of *C. churrovis* included maltotriitol, maltotriose, and melibiose. These metabolites were either detected at low levels or not detected in *A. robustus* and the media (Figure S2). Maltotriitol, a sugar alcohol known to inhibit bacterial metabolism in *Streptococcus mutans* [33], may help *C. churrovis* regulate microbial interactions or sequester nutrients in the native rumen. Maltotriose and melibiose are alpha-linked carbohydrate oligomers that are breakdown products of starch and glycogen, and their presence aligns with prior findings that the gut fungus *Neocallimastix frontalis* produces alpha-amylases that release such oligosaccharides [34]. Notably, maltotriose may contribute to *C. churrovis* biofilm formation, similar to how *Aureobasidium pullulans* polymerizes maltotriose to form the extracellular polysaccharide pullulan [35]. Unlike *A. robustus* and other rhizoidal fungi, *C. churrovis* may predominately depend on extracellular polymers to adhere to substrates and create biofilms [32,36,37]. Characterizing these polymers could advance biotechnological applications for *C. churrovis* and improve biofilm management for scaling up bioreactors. Meanwhile, melibiose is likely unusable by gut fungi [38] and may accumulate as a metabolic by-product. In the native rumen microbiome, metabolic by-products like melibiose may help shape community membership through cross-feeding interactions [39].

*A. robustus* and *C. churrovis* potentially concentrated multiple metabolites from the media. For example, they likely acquired monoaromatic compounds, including phloretic acid, isovanillic acid, and p-salicylic acid (Figure S2). In the native rumen, gut fungi degrade lignin and utilize these aromatic breakdown products for further metabolism [4]. Between the strains, *A. robustus* enriched more mannose, and *C. churrovis* enriched more glyceric acid (glycerate) and lactulose, as well as key components of gut fungal central metabolism, malic acid (malate), and fumaric acid (fumarate) (Figure S2) [31,32].

**Figure 2:**
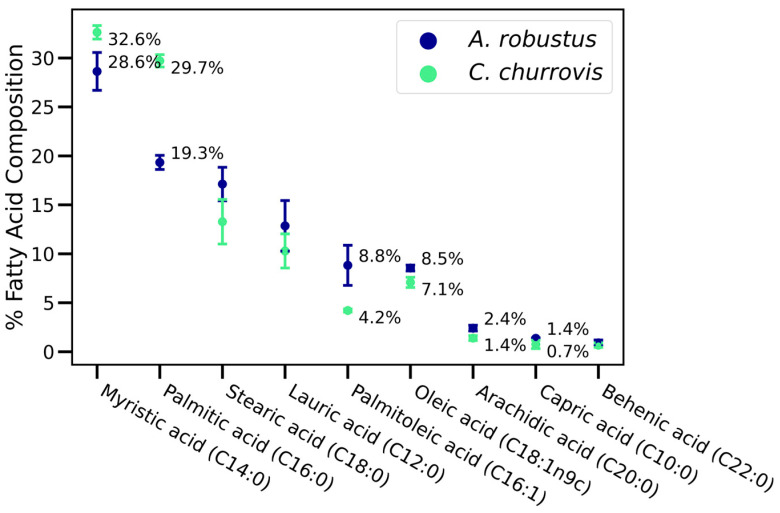
Several fatty acids were detected at different abundances between *A. robustus* and *C. churrovis*. Percent fatty acid composition was calculated as the ratio of fatty acid abundance to the total abundance of all fatty acids considered, with error bars indicating biological replicates. Only high-confidence matches (see Section 2) for fatty acids are displayed, and the omission of fatty acids does not indicate their absence in the samples.

Fatty acid profiling was performed to compare to previous GC-MS-based studies for anaerobic gut fungi [6,7,8,9,10] and to evaluate if the profiles provide taxonomic insight. In the animal gut, microbes metabolize essential fats from the host’s diet through hydrolysis or biohydrogenation. Because cell fatty acid composition reflects biosynthetic and metabolic capacity, fatty acid composition is a routine method to identify microbes, particularly prokaryotes [9,15,16]. In this study, the fatty acid profiles for *A. robustus* and *C. churrovis* were consistent with one previous report: myristic acid (C14:0) had the highest percent composition, with palmitic acid (C16:0) and stearic acid (C12:0) also relatively abundant [6] (Figure 2). Other studies reported conflicting results for genera. One study reported oleic acid (C18:1n9c) and palmitic acid (C16:0) as the most relatively abundant for *Caecomyces* sp. GMLF12 [10]. A second study reported arachidic acid (C20:0) and stearic acid (C18:0) as the most relatively abundant for *Caecomyces* sp. OF1 and lauric acid (C12:0) and heneicosanoic acid (C21:0, not considered in this study) were determined as the most abundant for *Anaeromyces mucronatus* sp. KF8, sp. JF1, and sp. ZF1 [9]. These discrepancies are tentatively due to cultivation conditions and strain-level metabolic differences. As noted by Kar et al., the variability in fatty acid compositions for anaerobic gut fungal genera limits the utility of fatty acid composition for robust taxonomic identification [6].

### 3.2. Genome Mining and Molecular Networking Highlight a Putative Terpene and Terpenoid Produced by Anaerobic Gut Fungi

Untargeted GC-MS profiling can expand our limited knowledge of anaerobic gut fungal secondary metabolites, particularly volatile terpenes and terpenoids, which have a broad range of applications in pharmaceuticals, fragrances, food, and biofuels [40]. Unlike most eukaryotes that biosynthesize oxygen-dependent sterols to modulate membrane fluidity [41], anaerobic eukaryotes cannot produce sterols and instead scavenge them from the environment or biosynthesize sterol surrogates, like the triterpenoid tetrahymanol [42,43,44,45,46,47]. In anaerobic gut fungi, tetrahymanol is thought to be produced from squalene via biosynthetic genes horizontally transferred from prokaryotes [7,19,20]. In this study, we putatively identified the expected squalene and tetrahymanol produced by *A. robustus* and *C. churrovis* and provide supporting evidence for annotating their biosynthetic genes.

In silico genome mining with antiSMASH v7 [48] predicts a terpene-like biosynthetic gene cluster conserved in *A. robustus*, *C. churrovis*, and other gut fungal strains (see Supplementary Materials and Methods). This conserved cluster contains a core biosynthetic gene annotated as squalene synthase (SQS) (TIGR01559), so its likely function is to biosynthesize squalene from two molecules of farnesyl diphosphate (Figure S3). In other anaerobic eukaryotes, like *Trimastix pyriformis*, squalene tetrahymanol cyclase (STC) cyclizes squalene to tetrahymanol. Based on a BLASTp comparison of the *T. pyriformis* STC gene (GenBank BAL49999) and previous phylogenetic analysis [19], several gut fungal strains encode likely STCs, which antiSMASH v7 fails to annotate (Figure S3).

**Figure 3:**
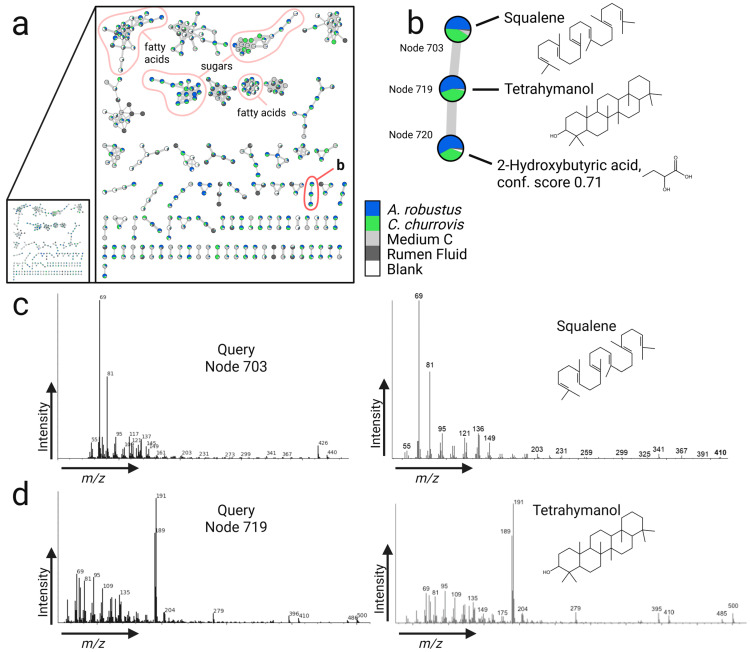
Metabolic networking highlights a putative terpenoid molecular family for *A. robustus* and *C. churrovis*. (**a**) The GNPS-generated Cytoscape network [17,18,26] shows molecular families of metabolites that were significantly more present in gut fungal samples compared to controls. Each node is a metabolite feature with a corresponding retention time and mass spectrum. Some nodes have putative compound identifications, based only on mass spectral matching to the GNPS GC-MS EI (gas chromatography electron ionization) mass spectral library (Supplementary Material 2). Edges, or lines between nodes in a molecular family, suggest compounds of similar structure. Node pie charts depict relative abundances of total ion chromatogram (TIC)-normalized peak heights across sample types. Some putative identifications for molecular families are labeled and outlined in red, including a putative terpenoid family (labeled “b”). (**b**) *A. robustus* and *C. churrovis* were highly represented in this molecular family putatively containing tetrahymanol and squalene, which are expected gut fungal products. (**c**) Squalene (reference spectrum from LIPID MAPS, https://www.lipidmaps.org/, accessed on 31 January 2025) and (**d**) tetrahymanol [20] were putatively identified by manual inspection of mass spectra. Conf. score = GNPS MQ score (modified cosine score). Created in BioRender. Butkovich, L. (2025) https://BioRender.com/58cmg7l.

The GNPS analysis and metabolic networking aided in identifying the putative gut fungal terpenoids squalene and tetrahymanol, which were more abundant in *A. robustus* and *C. churrovis* than the media (Figure 3 and Figure S4). From the initial spectral matching with GNPS reference libraries, node 719 matched to “C(14a)-Homo-27-norgammacer-14-ene,” or serratene, which is structurally similar to tetrahymanol, and node 703 matched to “Glutaric acid, 3-methylbut-2-en-1-yl dec-4-enyl ester,” which is structurally similar to squalene. Manual inspection of node 719 and node 703 verified that their mass spectra were more similar to tetrahymanol and squalene, respectively. From the proteomic analysis, putative SQS was detected in *A. robustus* (proteinID 221108) and *C. churrovis* (proteinID 465791), and STC was detected at low levels in *A. robustus* (proteinID 327690) and not detected in *C. churrovis* (proteinID 456585) (Table S1) [49]. Although proteomic signals for STC were weak or absent in the gut fungi, SQS and STC may have been more abundant earlier in culture growth.

Closer inspection of GC-MS data via the GNPS-based spectral library search and molecular networking suggests the classification of additional gut fungal metabolites not addressed in the NIST-based analysis, although for these putative identifications, positional isomers cannot be confidently distinguished. Multiple putatively identified metabolites were either biosynthesized, acquired by the breakdown of media components, or concentrated directly from the media by *A. robustus* and *C. churrovis*. These metabolites include alkaloids (2,3-dimethyl-4-nitroindole and 2-hydroxyquinoline), a neurotransmitter (gamma-aminobutyric acid, or GABA), a diterpenoid phytol (3,7,11,15-tetramethyl-1-hexadecen-3-ol), an aromatic compound (4,4-Bis(4-hydroxyphenyl)valeric acid or diphenolic acid), a sugar alcohol (iditol), and additional fatty acids ((Z)-15-octadecenoic acid, tricosanoic acid, phytanic acid, and icosanoic acid) (Figure S4). Maltotriose, detected in the analysis with NIST 14 and internal reference libraries, was also putatively identified in the GNPS analysis, providing supporting evidence for this compound identification. Based on their low abundances in the media, the putative 2-hydroxyquinoline and diphenolic acid are possible gut fungal products. In silico genome mining for anaerobic gut fungi suggests an abundance of biosynthetic gene clusters for polyketides and non-ribosomal peptides [2]. From our proteomic analysis, multiple predicted polyketide synthases and non-ribosomal peptide synthetases were detected in *A. robustus* and *C. churrovis* (Table S1). Tentatively, the corresponding products of these biosynthetic enzymes are among the metabolite features of this and other previously published untargeted metabolomic analyses for anaerobic gut fungi [2,5]. The GC-MS technique used in this study likely captured only smaller, sufficiently volatile non-ribosomal peptides and polyketides if present, possibly the metabolites putatively identified as 2-hydroxyquinoline and diphenolic acid.

## 4. Conclusions

In this study, untargeted GC-MS profiling revealed metabolite products of the anaerobic fungi *A. robustus* and *C. churrovis*, providing a foundational dataset to generate hypotheses and inform future research, including efforts to isolate and characterize novel metabolites. The detection of key metabolites, including secondary metabolites like tetrahymanol, indicate unique biosynthetic pathways and biological strategies that anaerobic fungi use to establish and proliferate in their native rumen environment. Strain-specific metabolites, such as 2,3-dihydroxyisovaleric acid in *A. robustus* and maltotriitol, maltotriose, and melibiose in *C. churrovis*, may inform metabolic strategies, although their production may be influenced by culture conditions or similarly produced by other gut fungal strains. Expanding this analysis to additional strains, media, and substrates would deepen our understanding of anaerobic gut fungal metabolism and enable secondary metabolism to be more accurately accounted for in genome-scale metabolic modeling efforts. Overall, our findings underscore the need for further exploration and validation of gut fungal biosynthetic pathways.

## Data Availability

The metabolomics data and metadata were deposited in the public repository MassIVE (MSV000097279). The GNPS job can be accessed at https://gnps.ucsd.edu/ProteoSAFe/status.jsp?task=662c0f27caa94ee894877ce8a136a7e1 (accessed on 17 December 2024).

## Supplementary Materials

The following supporting information can be downloaded at https://www.mdpi.com/article/10.3390/metabo15090578/s1. Supplementary material references are provided [49,50,51,52,53].

## Abbreviations

The following abbreviations are used in this manuscript:

GC-MS	gas chromatography–mass spectrometry
MPLEx	Metabolite, Protein, and Lipid Extraction
LC-MS/MS	liquid chromatography–tandem mass spectrometry
GNPS	Global Natural Products Social Molecular Networking
PBS	phosphate buffered saline
PNNL	Pacific Northwest National Laboratory
MSTFA	N-methyl-N-trimethylsilyltrifluoroacetamide
TMS	trimethylsilylated
FAMEs	fatty acid methyl esters
RI	retention index
pPCA	probabilistic principal component analysis
SQS	squalene synthase
STC	squalene tetrahymanol cyclase
antiSMASH	antibiotics and Secondary Metabolite Analysis Shell
TIC	total ion chromatogram
GABA	gamma-aminobutyric acid

## References

[1] Liggenstoffer A.S., Youssef N.H., Couger M.B., Elshahed M.S. (2010). Phylogenetic Diversity and Community Structure of Anaerobic Gut Fungi (Phylum Neocallimastigomycota) in Ruminant and Non-Ruminant Herbivores. ISME J..

[2] Swift C.L., Louie K.B., Bowen B.P., Olson H.M., Purvine S.O., Salamov A., Mondo S.J., Solomon K.V., Wright A.T., Northen T.R. (2021). Anaerobic Gut Fungi Are an Untapped Reservoir of Natural Products. Proc. Natl. Acad. Sci. USA.

[3] Solomon K.V., Haitjema C.H., Henske J.K., Gilmore S.P., Borges-Rivera D., Lipzen A., Brewer H.M., Purvine S.O., Wright A.T., Theodorou M.K. (2016). Early-Branching Gut Fungi Possess a Large, Comprehensive Array of Biomass-Degrading Enzymes. Science.

[4] Lankiewicz T.S., Choudhary H., Gao Y., Amer B., Lillington S.P., Leggieri P.A., Brown J.L., Swift C.L., Lipzen A., Na H. (2023). Lignin Deconstruction by Anaerobic Fungi. Nat. Microbiol..

[5] Swift C.L., Louie K.B., Bowen B.P., Hooker C.A., Solomon K.V., Singan V., Daum C., Pennacchio C.P., Barry K., Shutthanandan V. (2021). Cocultivation of Anaerobic Fungi with Rumen Bacteria Establishes an Antagonistic Relationship. mBio.

[6] Kar B., Özköse E., Ekinci M.S. (2021). The Comparisons of Fatty Acid Composition in Some Anaerobic Gut Fungi *Neocallimastix*, *Orpinomyces*, *Piromyces*, and *Caecomyces*. An. Acad. Bras. Cienc..

[7] Kemp P., Lander D.J., Orpin C.G. (1984). The Lipids of the Rumen Fungus *Piromonas Communis*. J. Gen. Microbiol..

[8] Body D.R., Bauchop T. (1985). Lipid Composition of an Obligately Anaerobic Fungus *Neocallimastix Frontalis* Isolated from a Bovine Rumen. Can. J. Microbiol..

[9] Koppová I., Novotná Z., Štrosová L., Fliegerová K. (2008). Analysis of Fatty Acid Composition of Anaerobic Rumen Fungi. Folia Microbiol..

[10] Comlekcioglu U., Ozkose E., Akyol I., Ekinci M.S. (2010). Fatty Acid Analysis of Anaerobic Ruminal Fungi *Neocallimastix*, *Caecomyces* and *Orpinomyces*. Int. J. Agric. Biol..

[11] Fiehn O. (2016). Metabolomics by Gas Chromatography-Mass Spectrometry: Combined Targeted and Untargeted Profiling. Curr. Protoc. Mol. Biol..

[12] Breton A., Bernalier A., Dusser M., Fonty G., Gaillard-Martinie B., Guillot J. (2006). *Anaeromyces Mucronatus* Nov. Gen., Nov. Sp. A New Strictly Anaerobic Rumen Fungus with Polycentric Thallus. FEMS Microbiol. Lett..

[13] Henske J.K., Gilmore S.P., Knop D., Cunningham F.J., Sexton J.A., Smallwood C.R., Shutthanandan V., Evans J.E., Theodorou M.K., O’Malley M.A. (2017). Transcriptomic Characterization of *Caecomyces Churrovis*: A Novel, Non-Rhizoid-Forming Lignocellulolytic Anaerobic Fungus. Biotechnol. Biofuels.

[14] Gold J.J., Brent Heath I., Bauchop T. (1988). Ultrastructural Description of a New Chytrid Genus of Caecum Anaerobe, *Caecomyces Equi* Gen. Nov., Sp. Nov., Assigned to the Neocallimasticaceae. Biosystems.

[15] Wauthoz P., Lioui M.E., Decallonne J. (1995). Gas Chromatographic Analysis of Cellular Fatty Acids in the Identification of Foodborne Bacteria. J. Food Prot..

[16] Zhang S., Zhu J. (2022). Untargeted Metabolomics Sensitively Differentiates Gut Bacterial Species in Single Culture and Co-Culture Systems. ACS Omega.

[17] Aksenov A.A., Laponogov I., Zhang Z., Doran S.L.F., Belluomo I., Veselkov D., Bittremieux W., Nothias L.F., Nothias-Esposito M., Maloney K.N. (2021). Auto-Deconvolution and Molecular Networking of Gas Chromatography–Mass Spectrometry Data. Nat. Biotechnol..

[18] Wang M., Carver J.J., Phelan V.V., Sanchez L.M., Garg N., Peng Y., Nguyen D.D., Watrous J., Kapono C.A., Luzzatto-Knaan T. (2016). Sharing and Community Curation of Mass Spectrometry Data with Global Natural Products Social Molecular Networking. Nat. Biotechnol..

[19] Murphy C.L., Youssef N.H., Hanafy R.A., Couger M.B., Stajich J.E., Wang Y., Baker K., Dagar S.S., Griffith G.W., Farag I.F. (2019). Horizontal Gene Transfer as an Indispensable Driver for Evolution of Neocallimastigomycota into a Distinct Gut-Dwelling Fungal Lineage. Appl. Environ. Microbiol..

[20] Takishita K., Chikaraishi Y., Leger M.M., Kim E., Yabuki A., Ohkouchi N., Roger A.J. (2012). Lateral Transfer of Tetrahymanol-Synthesizing Genes Has Allowed Multiple Diverse Eukaryote Lineages to Independently Adapt to Environments without Oxygen. Biol. Direct.

[21] Makkar H.P., McSweeney C.S. (2005). Methods in Gut Microbial Ecology for Ruminants.

[22] Nakayasu E., Nicora C., Sims A., Burnum-Johnson K., Kim Y.-M., Kyle J., Matzke M., Shukla A., Chu R., Schepmoes A.A. (2016). MPLEx: A Robust and Universal Protocol for Single-Sample Integrative Proteomic, Metabolomic, and Lipidomic Analyses. mSystems.

[23] Hiller K., Hangebrauk J., Jäger C., Spura J., Schreiber K., Schomburg D. (2009). Metabolite Detector: Comprehensive Analysis Tool for Targeted and Nontargeted GC/MS Based Metabolome Analysis. Anal. Chem..

[24] Kind T., Wohlgemuth G., Lee D.Y., Lu Y., Palazoglu M., Shahbaz S., Fiehn O. (2009). FiehnLib: Mass Spectral and Retention Index Libraries for Metabolomics Based on Quadrupole and Time-of-Flight Gas Chromatography/Mass Spectrometry. Anal. Chem..

[25] Lai Z., Tsugawa H., Wohlgemuth G., Mehta S., Mueller M., Zheng Y., Ogiwara A., Meissen J., Showalter M., Takeuchi K. (2018). Identifying Metabolites by Integrating Metabolome Databases with Mass Spectrometry Cheminformatics. Nat. Methods.

[26] Shannon P., Markiel A., Ozier O., Baliga N.S., Wang J.T., Ramage D., Amin N., Schwikowski B., Ideker T. (2003). Cytoscape: A Software Environment for Integrated Models of Biomolecular Interaction Networks. Genome Res..

[27] Haitjema C.H., Gilmore S.P., Henske J.K., Solomon K.V., De Groot R., Kuo A., Mondo S.J., Salamov A.A., LaButti K., Zhao Z. (2017). A Parts List for Fungal Cellulosomes Revealed by Comparative Genomics. Nat. Microbiol..

[28] Weber T., Blin K., Duddela S., Krug D., Kim H.U., Bruccoleri R., Lee S.Y., Fischbach M.A., Müller R., Wohlleben W. (2015). AntiSMASH 3.0-A Comprehensive Resource for the Genome Mining of Biosynthetic Gene Clusters. Nucleic Acids Res..

[29] Kim S., Pevzner P.A. (2014). MS-GF+ Makes Progress towards a Universal Database Search Tool for Proteomics. Nat. Commun..

[30] Pruett S.T., Bushnev A., Hagedorn K., Adiga M., Haynes C.A., Sullards M.C., Liotta D.C., Merrill A.H. (2008). Thematic Review Series: Sphingolipids. Biodiversity of Sphingoid Bases (“Sphingosines”) and Related Amino Alcohols. J. Lipid Res..

[31] Wilken S.E., Monk J.M., Leggieri P.A., Lawson C.E., Lankiewicz T.S., Seppälä S., Daum C.G., Jenkins J., Lipzen A.M., Mondo S.J. (2021). Experimentally Validated Reconstruction and Analysis of a Genome-Scale Metabolic Model of an Anaerobic Neocallimastigomycota Fungus. mSystems.

[32] Leggieri P.A., Valentine M.T., O’Malley M.A. (2022). Biofilm Disruption Enhances Growth Rate and Carbohydrate-Active Enzyme Production in Anaerobic Fungi. Bioresour. Technol..

[33] Würsch P., Koellreutter B. (1985). Maltotriitol Inhibition of Maltose Metabolism in Streptococcus Mutans via Maltose Transport, Amylomaltase and Phospho-α-Glucosidase Activities. Caries Res..

[34] Mountfort D., Asher R. (1988). Production of Alpha-Amylase by the Ruminal Anaerobic Fungus *Neocallimastix frontalis*. Appl. Environ. Microbiol..

[35] Wei X., Liu G.-L., Jia S.-L., Chi Z., Hu Z., Chi Z.-M. (2021). Pullulan Biosynthesis and Its Regulation in *Aureobasidium* spp.. Carbohydr. Polym..

[36] Brethauer S., Shahab R.L., Studer M.H. (2020). Impacts of Biofilms on the Conversion of Cellulose. Appl. Microbiol. Biotechnol..

[37] Chandrasekar P.H., Manavathu E.K. (2008). Do *Aspergillus* Species Produce Biofilm?. Future Microbiol..

[38] Phillips M.W., Gordon G.L.R. (1988). Sugar and Polysaccharide Fermentation by Rumen Anaerobic Fungi from Australia, Britain and New Zealand. Biosystems.

[39] Culp E.J., Goodman A.L. (2023). Cross-Feeding in the Gut Microbiome: Ecology and Mechanisms. Cell Host Microbe.

[40] Tetali S.D. (2019). Terpenes and Isoprenoids: A Wealth of Compounds for Global Use. Planta.

[41] Bui T.T., Suga K., Umakoshi H. (2016). Roles of Sterol Derivatives in Regulating the Properties of Phospholipid Bilayer Systems. Langmuir.

[42] Ourisson G., Rohmer M., Poralla K. (1987). Prokaryotic Hopanoids and Other Polyterpenoid Sterol Surrogates. Annu. Rev. Microbiol..

[43] Sáenz J.P., Grosser D., Bradley A.S., Lagny T.J., Lavrynenko O., Broda M., Simons K. (2015). Hopanoids as Functional Analogues of Cholesterol in Bacterial Membranes. Proc. Natl. Acad. Sci. USA.

[44] Nes W.D., Heftmann E. (1981). A Comparison of Triterpenoids with Steroids as Membrane Components. J. Nat. Prod..

[45] Gruninger R.J., Puniya A.K., Callaghan T.M., Edwards J.E., Youssef N., Dagar S.S., Fliegerova K., Griffith G.W., Forster R., Tsang A. (2014). Anaerobic Fungi (Phylum *Neocallimastigomycota*): Advances in Understanding Their Taxonomy, Life Cycle, Ecology, Role and Biotechnological Potential. FEMS Microbiol. Ecol..

[46] Youssef N.H., Couger M.B., Struchtemeyer C.G., Liggenstoffer A.S., Prade R.A., Najar F.Z., Atiyeh H.K., Wilkins M.R., Elshahed M.S. (2013). The Genome of the Anaerobic Fungus *Orpinomyces* Sp. Strain C1a Reveals the Unique Evolutionary History of a Remarkable Plant Biomass Degrader. Appl. Environ. Microbiol..

[47] Wiersma S.J., Mooiman C., Giera M., Pronk J.T. (2020). Squalene-Tetrahymanol Cyclase Expression Enables Sterol-Independent Growth of *Saccharomyces Cerevisiae*. Appl. Environ. Microbiol..

[48] Blin K., Shaw S., Augustijn H.E., Reitz Z.L., Biermann F., Alanjary M., Fetter A., Terlouw B.R., Metcalf W.W., Helfrich E.J.N. (2023). AntiSMASH 7.0: New and Improved Predictions for Detection, Regulation, Chemical Structures and Visualisation. Nucleic Acids Res..

[49] Grigoriev I.V., Nikitin R., Haridas S., Kuo A., Ohm R., Otillar R., Riley R., Salamov A., Zhao X., Korzeniewski F. (2014). MycoCosm Portal: Gearing up for 1000 Fungal Genomes. Nucleic Acids Res..

[50] Mondo S.J., Dannebaum R.O., Kuo R.C., Louie K.B., Bewick A.J., LaButti K., Haridas S., Kuo A., Salamov A., Ahrendt S.R. (2017). Widespread Adenine N6-Methylation of Active Genes in Fungi. Nat. Genet..

[51] Li Y., Li Y., Jin W., Sharpton T.J., Mackie R.I., Cann I., Cheng Y., Zhu W. (2019). Combined Genomic, Transcriptomic, Proteomic, and Physiological Characterization of the Growth of Pecoramyces Sp. F1 in Monoculture and Co-Culture with a Syntrophic Methanogen. Front. Microbiol..

[52] Enright A.J., Van Dongen S., Ouzounis C.A. (2002). An Efficient Algorithm for Large-Scale Detection of Protein Families. Nucleic Acids Res..

[53] Khaldi N., Seifuddin F.T., Turner G., Haft D., Nierman W.C., Wolfe K.H., Fedorova N.D. (2010). SMURF: Genomic Mapping of Fungal Secondary Metabolite Clusters. Fungal Genet. Biol..

