# Supplementary material for: Untargeted GC-MS Metabolic Profiling of Anaerobic Gut Fungi Reveals Putative Terpenoids and Strain-Specific Metabolites
Source: Metabolites. 2025 Aug 29;15(9):578. doi: 10.3390/metabo15090578 (PMC12471499; doi:10.3390/metabo15090578)
Supplement: Supplementary file 1 [file metabolites-15-00578-s001.zip › Supplementary Info.pdf]

**Supplementary Information: Untargeted GC-MS Metabolic Profiling of Anaerobic Gut Fungi Reveals Putative Terpenoids and Strain-Specific Metabolites**

Lazarina V. Butkovich[1], Candice L. Swift[1], Chaevien S. Clendinen[2,3], Heather M. Olson[2,3], Samuel O. Purvine[2,3], Oliver B. Vining[4], Michelle A. O'Malley[1]*
[1] Department of Chemical Engineering, University of California, Santa Barbara, Santa Barbara, CA 93106, USA
[2] Earth and Biological Sciences Division, Pacific Northwest National Laboratory, Richland, WA 99352, USA;
[3] Environmental Molecular Sciences Laboratory, Pacific Northwest National Laboratory, Richland, WA 99352, USA
[4] Institute for Collaborative Biotechnologies, University of California, Santa Barbara, CA 93106, USA

*Corresponding author

**Supplementary Material Descriptions**

**Supplementary Material 1**
Data are supplied for raw GC-MS data files processed with Metabolite Detector (log2-transformed and normalized to global median), and compounds were identified using the NIST 14 and internal reference libraries. High confidence matches (retention index match and >80% mass spectral match) are denoted in green, and low confidence matches (retention index match and either a <80% spectral match or a >80% spectral match to multiple compounds) are denoted in yellow. Metabolite abundances are provided in the following data columns: "AR" for *A. robustus* replicates, "CC" for *C. churrovis*, "MC" for Medium C, and "RF" for rumen fluid.

**Supplementary Material 2**
Data are supplied for raw GC-MS data files processed with MS-DIAL (v4.9) (normalized by total ion chromatogram (TIC)) and analyzed with GNPS library search and molecular networking. Feature IDs are denoted "shared name," which corresponds to Cytoscape feature node IDs and "Alignment_ID_MSDIAL." "Compound_Name_GNPS" denotes the best library match for a feature, and "MQScore_GNPS" denotes the MQ score (modified cosine score), a confidence metric for spectral matching. Data for metabolite abundances are provided for BLANK samples ("GCMS_BLANK_01_GCMS01_20201209", ("GCMS_BLANK_02_GCMS01_20201209", ("GCMS_BLANK_03_GCMS01_20201209"), a FAMES sample ("GCMS_FAMES_01_GCMS01_20201209"), *A. robustus* samples ("OMALL_RFS_AR_S4_#_M"), *C. churrovis* samples ("OMALL_RFS_CC#_M"), Medium C samples ("OMALL_RFS_MC#_M"), and rumen fluid samples ("OMALL_RFS_RF#_M"), where # = 1 to 4.

**Supplementary Material 3**
Processed proteomics data are supplied for *A. robustus* samples. The column labeled "Sequence" describes the sequence for each detected protein, "ProteinName." Data columns "OMall_RFS_Ar_S4_#_P," where # = 1 to 4, list spectral observation counts for each protein across *A. robustus* replicates.

**Supplementary Material 4**
Analogous to Supplementary Material 3, processed proteomics data are supplied for *C. churrovis* samples. Data columns "OMall_RFS_Cc#_P," where # = 1 to 4, list spectral observation counts for each protein across *C. churrovis* replicates.

**Supplementary Materials and Methods**

*Statistical Analysis with Compound Identifications from NIST 14 and Internal Reference Libraries*

Raw GC-MS data files in Agilent .D format were converted to netCDF format using Agilent Chemistation. Files were then converted to binary files using Metabolite Detector. The formatted GC-MS data files were processed using Metabolite Detector software, version 2.5 beta [23]. Retention indices of detected metabolites were calculated based on analysis of the standard mixture of fatty acid methyl esters (FAMEs) followed by chromatographic deconvolution and alignment. Metabolites were initially identified by matching experimental spectra to an augmented version of FiehnLib [24]. All FiehnLib metabolite identifications were then manually validated with the NIST 14 GC–MS library. The summed abundances of the three most abundant fragment ions of each identified metabolite were integrated across the GC elution profile (automatically determined by Metabolite Detector). Fragment ions due to trimethylsilylation (*m/z* 73 and 147) were excluded from the determination of metabolite abundance. Features resulting from GC column bleeding were removed from the data before further data processing and analysis. For statistical analysis of compound identifications using NIST 14, the processed GC-MS data was further analyzed in Matlab 2020b (Supplementary Material 1). All zeros were replaced with NaN, and the peak area data was log2-transformed and globally normalized to the median for downstream statistical analysis. Any given metabolite was only considered to be in a sample group if it was present in 3 or more replicates. If not, the metabolite was considered 'absent' (zero) in all replicates for that group.

Multiple data visualization techniques were applied to the processed GC-MS data. Probabilistic principal components analysis (pPCA) and spearman rank correlation were performed (Figure S1), and metabolite abundance bar charts (Figure S2) were generated. To calculate log2 fold-change values and perform student's t-tests for raw p-values for the volcano plot (Figure 1), zero values were substitute with 1/5 the value of the minimum non-zero value in the dataset.

*Molecular Networking and Additional Putative Compound Identification with GNPS*

Raw GC-MS .mzML files were processed with MS-DIAL (v4.9) [25] to detect peaks, align peaks by Kovats retention index, filter for peak quality, and normalize peak heights by TIC. Default settings were used except for the following: the ionization type is hard ionization (GC-MS), the retention time range is 0 to 38 min, the minimum peak height for peak detection is 1E5, and peaks were filtered for those detected in at least 50% of replicates in any sample group. The GNPS GC-MS EI data analysis pipeline (accessed on 17 December 2024) was used for molecular networking and putative compound identifications via library search [17,18] (Supplementary Material 2). Default settings were used at all steps, except fragment ion mass tolerance was set to 1.0 Da, since single quadrupole mass spectrometry data is considered low-resolution. Custom python scripts (https://github.com/O-Malley-Lab/GF_GCMS_data_analysis, accessed on 26 August 2025) were written for additional data analysis, such as generating log2 fold-change and p-values. Statistical analysis was performed for differential detection of metabolite features processed with MS-DIAL. Between two sample groups, metabolite features were defined as significantly different in abundance (average TIC-normalized peak height) using the following cutoffs: (1) $q < 0.05$, (2) log2 fold-change > 3, and (3) average log10 raw peak intensity > 5, to ignore features with relatively low abundance. Heatmaps were generated for putatively identified metabolites by computing z-scores with SciPy (Figure 1 a-c).

In Silico *Genome Mining and Biosynthetic Gene Annotations for Squalene and Tetrahymanol*

Biosynthetic gene clusters, including a terpene cluster with squalene synthase (SQS), were predicted by antiSMASH v7 [48] for eight anaerobic gut fungal genomes available in the Joint Genome Institute (JGI) MycoCosm portal [49] (https://mycocosm.jgi.doe.gov/, accessed on 23 January 2025): *Anaeromyces robustus* S4, *Caecomyces churrovis* A, *Neocallimastix californiae* G1, *Neocalllimastix lanati*, *Neocallimastix* sp. Gf-Ma3-1, *Neocallimastix* sp. WI3-B, *Piromyces finnis*, and *Piromyces* sp. UH3-1 [3,27,31,50]. Genome assembly (FASTA format) and annotation (GFF3 format) files were submitted to the

antiSMASH v7 server (accessed on 11 August 2024) to perform *in silico* genome mining, and default parameters were used [48]. Squalene tetrahymanol cyclase (STC) function was putatively annotated for multiple anaerobic gut fungal genomes by BLASTp comparison to the STC gene from *T. pyriformis* (GenBank BAL49999). For synteny analysis of SQS and STC genes across twelve strains of anaerobic gut fungi, the JGI MycoCosm portal was used to generate Markov Cluster Algorithm (MCL cluster) plots [3,13,27,31,46,49-52].

**Supplementary Figures**

a

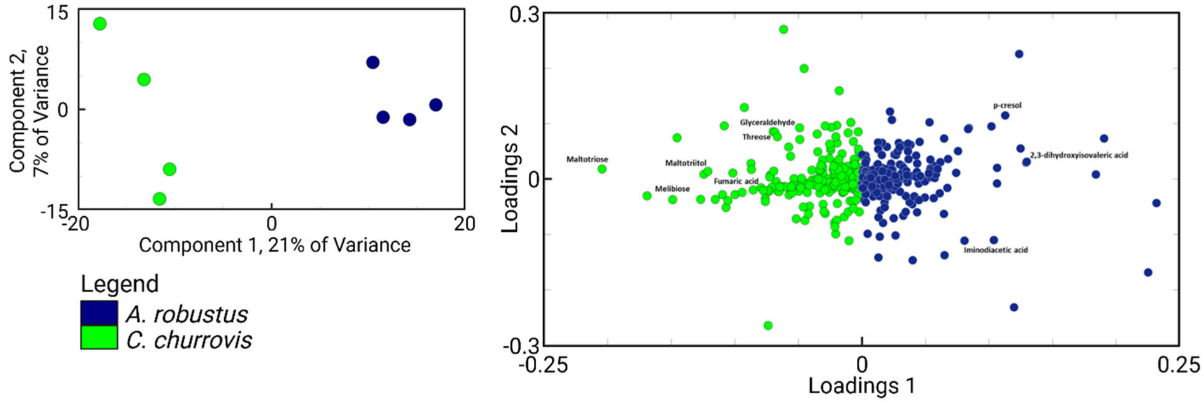


b

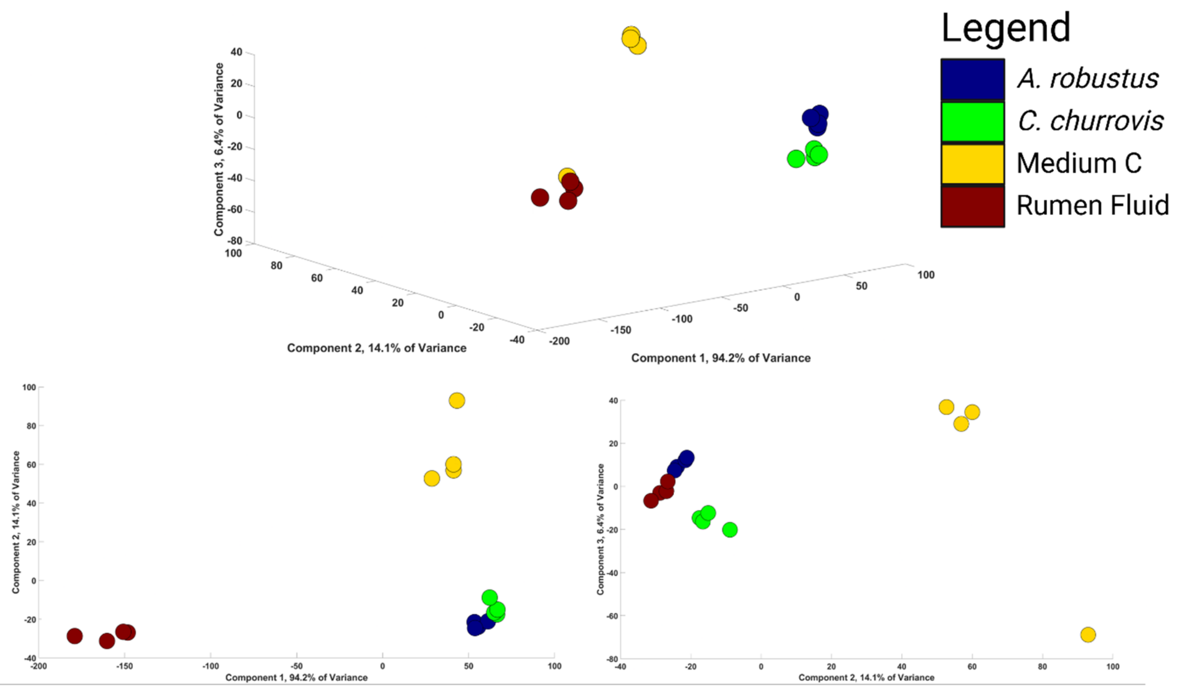


c

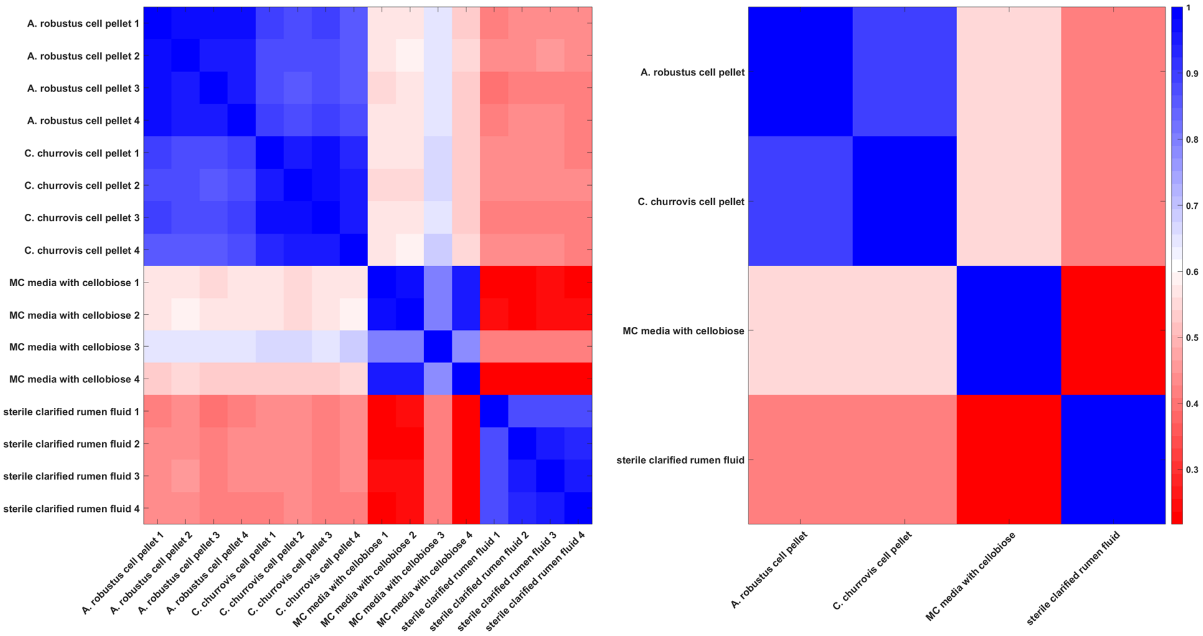


**Figure S1. GC-MS profiling differentiates *A. robustus*, *C. churrovis*, Medium C, and rumen fluid samples.** (a-b) Probabilistic principal component analysis (pPCA) and (c) spearman rank correlations showed expected differentiation of the different sample types. The pPCA for metabolite features (known and unknown) differentiates *A. robustus* and *C. churrovis* samples, and pPCA loadings indicate metabolites that contribute most significantly to sample group differences. The gut fungal strains cluster more closely with each other than to Medium C or rumen fluid, and Medium C replicate #3 clusters less well with its sample group. Figure was created with BioRender.com (accessed on 14 August 2025).

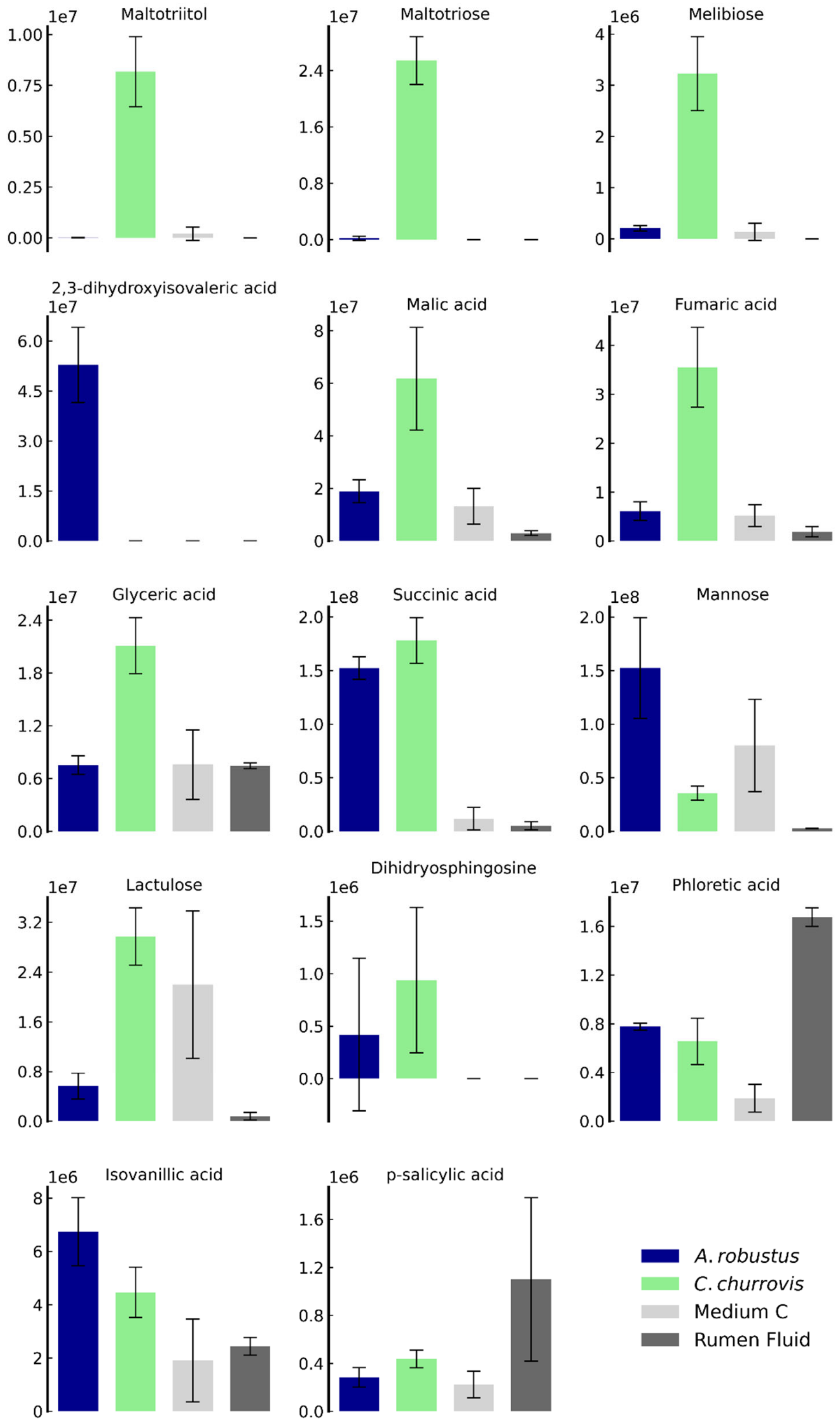


**Figure S2. Comparison of metabolite abundances indicates likely gut fungal products.** Multiple metabolites were identified by retention index and mass spectral matching to the NIST 14 and internal

reference libraries. The bar plots depict metabolites that were either produced by the gut fungi or acquired and concentrated from the media.

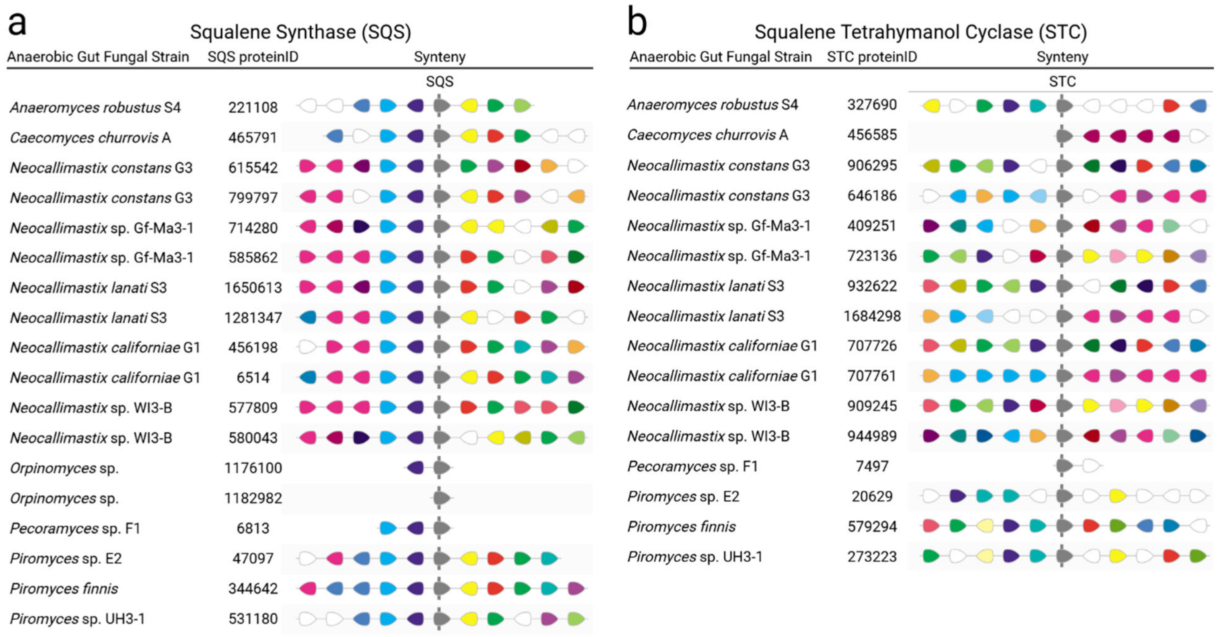


**Figure S3. Synteny analysis across twelve anaerobic gut fungal strains reveals conserved (a) squalene synthase (SQS) and (b) squalene tetrahymanol cyclase (STC).** Synteny analysis was performed on putative SQS and STC genes with physically co-localized genes of anaerobic gut fungal strains (see Section 2). Across strains, the putative SQS and STC genes did not physically co-localize with each other. Plot colors indicate same predicted gene functions, although colors between part a and part b do not correlate. (A) Based on antiSMASH v7 [48], *A. robustus* proteinID 221108 (e-value 3.6e-119) and *C. churrovis* proteinID 465791 (e-value 7.5e-121) likely encode SQSs (TIGR01559), where proteinIDs refer to gene identifiers in the JGI MycoCosm portal [49]. Genes co-localized to SQS are fairly conserved across strains. (B) Based on BLASTp comparison of the *T. pyriformis* STC gene (GenBank BAL49999), *A. robustus* proteinID 327690 (47.3% identity, 59.1% subject coverage, e-value 9.3E-42) and *C. churrovis* proteinID 456585 (44.1% identity, 62.2% subject coverage, e-value 3.4E-39) likely encode STCs. Genes co-localized to STC are not well-conserved between strains. Strains in the *Neocallimastix* genus appear to possess two SQS genes and two STC genes. A STC gene was not putatively identified in *Orpinomyces* sp., potentially due to high fragmentation in the assembled genome. Plots were acquired from the JGI MycoCosm (https://mycocosm.jgi.doe.gov, accessed on 23 January 2025) [49], and the figure was created with BioRender.com (accessed on 21 March 2025).

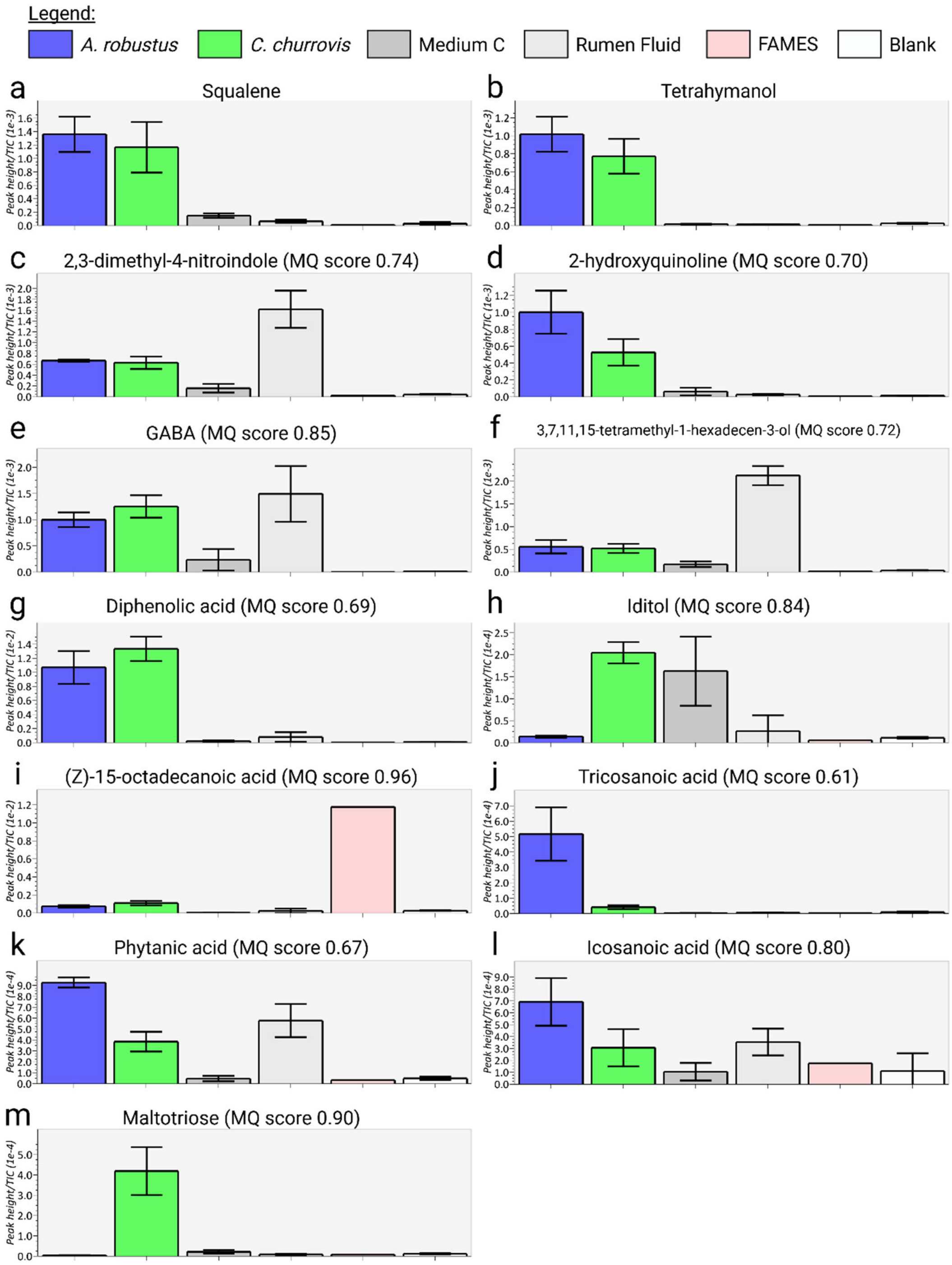


**Figure S4. Multiple metabolites putatively identified by mass spectral matching in GNPS were either produced by the gut fungi or acquired and concentrated from the media.** MQ scores refer to modified cosine scores generated in GNPS for spectral matching confidence. Matches with MQ score <0.90 are generally not correct for exact chemical structures but indicate structural similarity between query and library spectra. Therefore, these matches predominately suggest class-level information for detected metabolite features. FAMES = fatty acid methyl esters (C8-C28). Plots were created with MS-DIAL, and the figure was created with BioRender.com (accessed on 14 August 2025).

a. *A. robustus*

| JGI MycoCosm Biosynthetic Gene Cluster | antiSMASH v3 Biosynthetic Gene Cluster Name [2] | Proteomics ID | proteinID | Predicted Secondary Metabolite Class | Detected in #/4 replicates |
|---|---|---|---|---|---|
| Anasp1.2 | Anasp_cluster13 | Anasp1.2 | 245209 | PKS | 4/4 |
| Anasp1.6 | - | Anasp1.6 | 295066 | PKS-like | 1/4 |
| Anasp1.9 | Anasp_cluster27 | ctg279_allorf000290 | 296037 | NRPS | 2/4 |
| Anasp1.13 | Anasp_cluster28 | ctg305_allorf000001 | 271337 | NRPS | 1/4 |
| Anasp1.14 | Anasp_cluster1 | ctg5_allorf000927 | 289077 | PKS | 4/4 |
| Anasp1.16 | Anasp_cluster20 | ctg182_allorf000069 | 294553 | NRPS | 3/4 |
| Anasp1.19 | Anasp_cluster34 | ctg443_allorf000145 | 297598 | NRPS | 4/4 |
| Anasp1.22 | Anasp_cluster15 | ctg140_allorf000950 | 245515 | NRPS | 2/4 |
| Anasp1.24 | Anasp_cluster6 | ctg58_allorf000922 | 291548 | NRPS-like | 1/4 |
| Anasp1.26 | Anasp_cluster40 | ctg540_allorf000242 | 330657 | NRPS | 4/4 |
| Anasp1.31 | - | Anasp1.31 | 271916 | NRPS | 4/4 |
| Anasp1.35 | Anasp_cluster21 | ctg194_allorf000529 | 269811 | NRPS | 2/4 |
| Anasp1.36 | - | Anasp1.36 | 206444 | PKS-like | 1/4 |
| Anasp1.37 | Anasp_cluster26 | Anasp1.37 and ctg258_allorf000323 | 248107 | PKS | 4/4 |
| Anasp1.39 | Anasp_cluster37 | Anasp1.39 and ctg483_orf00001 | 212224 | PKS | 4/4 |
| Anasp1.43 | Anasp_cluster29 | ctg326_allorf000118 | 296554 | NRPS | 1/4 |
| - | Anasp_cluster19 | ctg155_allorf000129 | 221108 | Terpene (SQS) | 3/4 |
| - | - | Locus1837v1 rpkm35.40_Frame=1 | 327690 | Terpene (STC) | 1/4 |
| - | Anasp_cluster42 | ctg722_allorf000013 | 273575 | Cluster Finder saccharide | 2/4 |

b. *C. churrovis*

| JGI MycoCosm Biosynthetic Gene Cluster | antiSMASHv3 Biosynthetic Gene Cluster Name [2] | Proteomics ID | proteinID | Predicted Secondary Metabolite Class | Detected in #/4 replicates |
|---|---|---|---|---|---|
| Caecom1.5 | Caecom_cluster8 | Caecom_cluster8 | 17094 | PKS | 4/4 |
| Caecom1.7 | Caecom_cluster2 | Caecom_cluster2 | 489529 | PKS | 4/4 |
| Caecom1.9 | Caecom_cluster19 | Caecom_cluster19 | 554372 | NRPS | 4/4 |
| Caecom1.10 | Caecom_cluster11 | Caecom_cluster11 | 27727 | PKS | 3/4 |
| Caecom1.13 | Caecom_cluster9 | Caecom_cluster9 | 547760 | PKS | 4/4 |
| Caecom1.14 | Caecom_cluster17 | Caecom_cluster17 | 496832 | PKS | 4/4 |
| - | Caecom_cluster18 | Caecom_cluster18 | 553382 | NRPS-like | 4/4 |
| - | Caecom_cluster22 | Caecom_cluster22 | 465791 | Terpene (SQS) | 4/4 |
| - | - | Not detected in proteomics data | 456585 | Terpene (STC) | 0/4 |

**Table S1. Proteomics analysis detected predicted secondary metabolite biosynthetic enzymes in (a) *A. robustus* and (b) *C. churrovis*.** Previously, Swift et al. predicted biosynthetic gene clusters and their core biosynthetic genes for *A. robustus* and *C. churrovis*, using antiSMASHv3 (accessed on 9 February 2020) [28] and the SMURF algorithm [53], accessible from the JGI MycoCosm portal [2,49]. Many predicted PKSs and

NRPSs were at least lowly detected in all replicates for *A. robustus* and *C. churrovis*; tentatively some biosynthetic products were sufficiently volatile to detect with the GC-MS profiling and would be included in the set of unidentified, gut fungal product metabolites. Squalene synthase (SQS) was proteomically detected in *A. robustus* and *C. churrovis* samples, and squalene tetrahymanol cyclase (STC) was proteomically detected in only one *A. robustus* replicate. Proteomics IDs refer to the corresponding entry names in Supplementary Material 3 and 4. PKS = polyketide synthase, NRPS = non-ribosomal peptide synthetase, SQS = squalene synthase, STC = squalene-tetrahymanol cyclase.
